# The Impact of a Sibling's Death in Intensive Care Unit: Are We Doing Enough to Help Them?

**DOI:** 10.7759/cureus.2518

**Published:** 2018-04-22

**Authors:** Shaheen Sombans, Kamleshun Ramphul, Ruhi Sonaye

**Affiliations:** 1 Bharati Vidyapeeth Deemed University Medical College and Hospital, Sangli, Maharashtra, IND; 2 Department of Pediatrics, Shanghai Xin Hua Hospital Affiliated to Shanghai Jiao Tong University School of Medicine, Shanghai, CHN

**Keywords:** grief, emotional, intensive care unit, sibling

## Abstract

As physicians, we have lost many children who were under our care despite our best efforts. For most of us, after breaking the news to the family, we move on to treat our next patient who needs help. However, the family and most importantly the siblings have a harder time. The aim of this report is to try to understand how they cope with the loss of a sibling who was previously in intensive care unit.

## Editorial

Having worked in the Neonatal Intensive Care Unit (NICU), the Pediatric Intensive Care Unit and the Emergency Department, we have come across many cases where despite multiple attempts and efforts we lost the patient. After breaking the news to the family, we, as physicians, are usually done with our roles; but is it not our duty to provide additional care for the siblings?

To answer this question, we decided to go through different research articles that investigated the impact of such major event on the siblings. Brooten et al. studied the question and they found out that most adolescents were shocked and in disbelief after a sibling’s death. Eighty percent were told about the cause of the death and getting through burials was a very difficult phase for them. From seven to 13 months after the death, most experienced a rise in the fear of losing someone close and the thoughts of dying and girls were more affected than boys. Adolescents started being more considerate to their loved ones and showed more maturity by 13 months. Some also reported a sense of abandonment from their friends after the tragic event [1].

Many NICUs have different approaches to exposing siblings to their sick brother/sister [2]. A study done by Fanos et al. showed that most siblings acknowledged that they wished they could be involved with their sibling through that hard phase of life and they cherished any moment they got to spend with them while they were in intensive care [3]. In a paper published by Sandler et al., one such sibling described that although the death of her baby sister left her sad, shocked and mad, the only good memory she had from that experience was when she got to hold her baby sister in her arms. She dealt with the grief by talking about her baby sister with her parents and together they even wrote a song to honor her memory [4].

A wider survey was carried out by Foster et al. in 2012 where they included 40 families and 69% of the participants confirmed that there were changes in the siblings, some in terms of personality, school work, goals, life perspectives, activities and interest. Forty-seven percent said that they noticed changes in the relationship with family members and peers, while only 21% showed no changes attributed to death [5].

It seems undeniable that most siblings will not be left unaffected by a loss of their sibling. Sadly, not all hospitals provide appropriate follow-ups for the families in terms of psychological help and support. The ideal situation would be to create a follow-up program with a trained psychiatrist for the family, each interviewed alone at first, then as a family, on how they are dealing with the loss. Most developing and underdeveloped countries do not have support groups where family members who went through the same traumatic experience can meet and be each other’s support. Major hospitals in developing countries such as India and Mauritius should follow the path and example of many major health care centres and consider such alternatives to provide a better care for both the patients and their loved ones. Allowing them to open up to someone they can talk to and seek help from, will hopefully ease the pain they feel.
